# Self-Jumping of a Liquid Crystal Elastomer Balloon under Steady Illumination

**DOI:** 10.3390/polym14142770

**Published:** 2022-07-06

**Authors:** Dali Ge, Jielin Jin, Yuntong Dai, Peibao Xu, Kai Li

**Affiliations:** 1 School of Civil Engineering, Anhui Jianzhu University, Hefei 230601, China; dalige@ahjzu.edu.cn (D.G.); jinjielinjll@163.com (J.J.); daiytmechanics@ahjzu.edu.cn (Y.D.); peibaoxu@ahjzu.edu.cn (P.X.); 2 Institute of Advanced Technology, University of Science and Technology of China, Hefei 230001, China

**Keywords:** balloon, self-oscillation, jump, optically-responsive, liquid crystal elastomers

## Abstract

Self-oscillation capable of maintaining periodic motion upon constant stimulus has potential applications in the fields of autonomous robotics, energy-generation devices, mechano-logistic devices, sensors, and so on. Inspired by the active jumping of kangaroos and frogs in nature, we proposed a self-jumping liquid crystal elastomer (LCE) balloon under steady illumination. Based on the balloon contact model and dynamic LCE model, a nonlinear dynamic model of a self-jumping LCE balloon under steady illumination was formulated and numerically calculated by the Runge–Kutta method. The results indicated that there exist two typical motion regimes for LCE balloon under steady illumination: the static regime and the self-jumping regime. The self-jumping of LCE balloon originates from its expansion during contact with a rigid surface, and the self-jumping can be maintained by absorbing light energy to compensate for the damping dissipation. In addition, the critical conditions for triggering self-jumping and the effects of several key system parameters on its frequency and amplitude were investigated in detail. The self-jumping LCE hollow balloon with larger internal space has greater potential to carry goods or equipment, and may open a new insight into the development of mobile robotics, soft robotics, sensors, controlled drug delivery, and other miniature device applications.

## 1. Introduction

Oscillations are ubiquitous in nature, from the cell division of organisms to the rotation of planets around the sun, and from the alternation of day and night to the change of seasons. Mechanical oscillations are periodic energy conversion between potential energy and kinetic energy, and they usually rely on external alternating excitation to maintain the continuous motion of the system with damping dissipation [1]. In contrast, self-sustained oscillation is a phenomenon where an object sustains periodic motion upon a constant stimulus; thus, no additional complex human controls and portable batteries are required [2,3,4], and its frequency is often determined by its own characteristics [5]. In addition, self-sustained oscillation generally has good robustness [6]. Therefore, self-oscillation is of particular interest to scientists due to its great potential in various fields, such as soft robotics [7,8,9], energy-harvesters [10,11], mechano-logistic devices [12], sensors [13], and so on.

In recent years, various self-oscillating systems based on diverse stimuli-responsive materials are reported, such as hydrogels [14,15], dielectric elastomers [16], ionic gels [17], liquid crystal elastomers (LCEs) [7,18,19,20,21], and thermally responsive polymer materials [22], etc. Furthermore, a variety of self-sustained motion modes have been constructed, such as bending [23,24,25,26], buckling [27,28,29,30], torsion [31,32], stretching and shrinking [33,34], rolling [35,36], swimming [9], swinging [37,38], vibration [39,40,41], jumping [42,43,44], rotation [45], eversion or inversion [46,47], and even synchronized motion of several coupled self-oscillators [48]. These self-sustained motions often originate from nonlinear feedback mechanisms including self-shadowing [3,27,28], coupling of liquid volatilization and membrane deformation [49], coupling mechanism among air expansion and liquid column movement [50], and coupling of plate buckling and chemical reaction [18].

Among the different stimuli, light is the most favorable stimulus, which has the unique advantages of sustainability, precise controllability, and contactless driving [51,52]. As an important optically responsive material, the liquid crystal elastomer is synthesized by a composition of rodlike mesogenic monomers and backbones or side chains of flexible cross-linked polymers, and it combines rubber elasticity with liquid crystalline anisotropy to produce exceptional physical and optical properties [53,54,55,56,57,58]. When stimulated by external fields, such as light, heat, electricity, and magnetism, liquid crystal monomer molecules can change their configurations due to its rotation or phase transition, which induces macroscopic deformation [59]. Among various and effective stimuli in the LCE systems, light stimulus is more convenient to induce self-feedback to achieve self-sustained oscillation [43]. Recently, light-fueled self-oscillation based on LCE has attracted the attention of many scientists, and a large number of light-fueled self-oscillating systems based on LCE have been developed [30,43,44,59,60].

Although some self-oscillating systems based on LCE materials have been constructed, there is still a need to develop more modes of self-oscillating systems. The broader the range of available oscillation modes, the more versatile the autonomous devices one can potentially construct. As a common movement mode, jumping has remarkable performances, including ultrafast obstacle crossing, sudden energy release, and adaptability to complex terrain [61,62,63,64], and it can be a new way to produce reliable self-sustained oscillation [42,43,44]. Inspired by the active jumping of kangaroos and frogs in nature, in this paper, we proposed a self-sustained jumping LCE balloon under steady illumination, and investigated its dynamical behaviors of self-jumping. Different from a solid ball [44], the hollow LCE balloon with larger internal space has greater potential to carry goods or equipment. We expect that this study can provide new insights into understanding of self-oscillation phenomenon and promote the development of mobile robotics, soft robotics, sensors, controlled drug delivery, and other miniature device applications.

This paper is organized as follows. In Section 2, based on balloon contact model and dynamic LCE model, a nonlinear dynamic model of a self-jumping LCE balloon under steady illumination is formulated. In Section 3, the dynamic jumping of an LCE balloon under steady illumination is numerically calculated by the Runge–Kutta method. Two motion regimes of the LCE balloon under steady illumination are studied, and the mechanism of self-jumping is revealed in detail. In Section 4, the trigger conditions for self-jumping and the effects of various system parameters on frequency and amplitude are investigated. Finally, the paper concludes with a short summary in Section 5. 

## 2. Model and Formulation

In this section, a theoretical model is formulated for a self-jumping LCE balloon based on the balloon contact model and dynamic LCE model, including the dynamic of the jumping balloon, evolution of the number fraction in the LCE balloon, nondimensionalization of the system parameters, and the solution method of the differential governing equations with variable coefficients. 

### 2.1. Dynamic of the Spherical LCE Balloon

**Figure 1:**
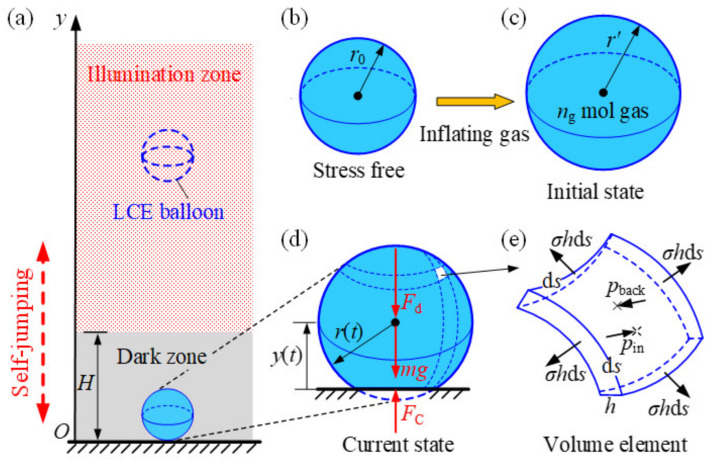
(**a**) Schematics of a self-jumping spherical LCE balloon under steady illumination. The radius $r\left(t\right)$ of the LCE balloon varies with time because of the light-driven contraction of the LCE membrane. (**b**) Reference state of the LCE balloon in the stress-free state. (**c**) Initial state of the LCE balloon inflated by gas. (**d**) The LCE balloon contacting rigid surface in dark zone is subjected to gravity mg, damping forces $F_{d}$, and contact force $F_{C}$. (**e**) Volume element. The LCE balloon can self-jump under steady illumination due to the expansion during contact with the rigid surface in the dark zone.

Figure 1 sketches an optically responsive LCE balloon with radius $r_{0}$ and membrane thickness $h_{0}$ in a stress-free state, which is capable of self-sustained jumping under steady illumination. The coordinate axis y along the vertical direction is introduced to describe the center position of the LCE balloon, and the origin O is fixed at the bottom of the rigid surface. The azobenzene liquid crystal molecules in the membrane of the inflated LCE balloon are parallel to its tangent plane. When the LCE balloon is in the illumination zone, the azobenzene liquid crystal molecules transform from the straight trans state to bent cis state, and thus the membrane shrinks in plane and expands in the thickness direction. We assume that the LCE material is incompressible and the volume of LCE membrane $V_{m}=4\pi r_{0}^{2}h_{0}$ is constant. Inflated by the gas of amount of substance $n_{g}$, the radius of LCE balloon is enlarged to r′ at the equilibrium state, which is set as the initial state as shown in Figure 1c. In this state, the inflated LCE balloon is initially released at the position $y_{0}$ in the illumination zone, and the LCE balloon drops from illumination zone ($y\left(t\right)>H$) to dark zone ($y\left(t\right)<H$), as shown in Figure 1a. In the illumination zone, the radius $r\left(t\right)$ of LCE balloon decreases and its membrane thickness $h\left(t\right)$ increases due to the light-driven contraction of LCE membrane. In the dark zone, its radius $r\left(t\right)$ increases and thickness $h\left(t\right)$ decreases with time due to the deformation recovery of the LCE membrane. Considering that the thickness is much smaller than the radius, we can calculate the membrane thickness as $h\left(t\right)=\frac{V_{m}}{4\pi r^{2}}$ due to the incompressibility of the LCE material.

During movement, the LCE balloon is subjected to gravity mg and damping forces $F_{d}$, which is assumed to be proportional to its velocity for simplicity. When contacting the rigid surface in the dark zone, the LCE balloon is also subjected to contact force $F_{C}$, as shown in Figure 1d. Therefore, the corresponding nonlinear dynamic governing equation of the LCE balloon can be given as follows

(1) $$-mg+F_{C}-\beta \dot{y}=m\ddot{y}$$

where g is gravitational acceleration, β is the damping coefficient, m is the mass of the LCE balloon, and $\dot{y}$ and $\ddot{y}$ are its velocity and acceleration, respectively. 

When the deformable and elastomeric balloon hits the flat and rigid surface, the balloon is pushed with a contact force $F_{C}$ from rigid surface [65], as shown in Figure 1d. Considering the elasticity of membrane and compression of internal ideal gas, and omitting the adhesion energy between the balloon and rigid surface, the contact force $F_{C}$ can be expressed by the balloon contact model as [65]

(2) $$F_{C}=0\;\mathrm{for}\;y\left(t\right)>r\left(t\right),$$

(3) $$F_{C}=\frac{3V_{g}\left(p_{\mathrm{in}}-p_{\mathrm{am}}\right)\left(r^{2}-y^{2}\right)}{2\left[2r^{3}+r\left(r^{2}-y^{2}\right)\left(1-8\lambda \right)\right]}\;\mathrm{for}\;y\left(t\right)\leq r\left(t\right),$$

where E and υ are the Young’s modulus and Poisson’s ratio, respectively, $\lambda =\frac{3rp_{\mathrm{in}}}{16\left(2E_{\mathrm{eff}}h+3rp_{\mathrm{in}}\right)}$ is a constant for the given balloon (at a given temperature and pressure), $p_{\mathrm{am}}$ is the ambient pressure, and $p_{\mathrm{in}}$ is the internal pressure. 

In Equation (3), the internal pressure $p_{\mathrm{in}}$ is generally related to the radius $r\left(t\right)$ of the balloon. For simplicity, the gas inside the balloon is assumed to be ideal gas with equation of state

(4) $$p_{\mathrm{in}}V_{g}=n_{g}RT,$$

where $V_{g}=\frac{4}{3}\pi r{\left(t\right)}^{3}$ is the instantaneous gas volume, R is the ideal gas constant, and T is the thermodynamic temperature of the ideal gas.

To determine the radius of the balloon, we neglect the effect of gravity and damping force on the deformation of the balloon; a spherical shell volume element with edge length ds of the LCE balloon is shown in Figure 1e. The equilibrium equation of the volume element in the normal direction is given as follows

(5) $$4\sigma hds\cdot \frac{1}{2}\frac{ds}{r}+p_{\mathrm{am}}{\left(ds\right)}^{2}-p_{\mathrm{in}}{\left(ds\right)}^{2}=0,$$

where $4\sigma hds\cdot \frac{1}{2}\frac{ds}{r}$ is the normal component of the tensile force on the LCE balloon. The principal stress σ can be derived by $\sigma =E_{\mathrm{eff}}\varepsilon$, where $E_{\mathrm{eff}}=\frac{1}{1-\upsilon }E$ is the effective elastic modulus of the equiaxial stress state, and $\varepsilon =\frac{r-r_{0}\left(1+\varepsilon_{L}\right)}{r_{0}\left(1+\varepsilon_{L}\right)}$ is the effective elastic strain induced by light-driven contraction $\varepsilon_{L}$ of the LCE membrane. Combined with Equation (4), Equation (5) can be rewritten as

(6) $$\frac{3n_{g}RT}{4\pi r^{3}}-\frac{E_{\mathrm{eff}}\left[r-r_{0}\left(1+\varepsilon_{L}\right)\right]}{r_{0}\left(1+\varepsilon_{L}\right)}\frac{2V_{m}}{4\pi r^{3}}-p_{\mathrm{am}}=0.$$

Equation (6) determines the radius $r\left(t\right)$ of the balloon for a given light-driven contraction $\varepsilon_{L}\left(t\right)$ of the LCE membrane. 

### 2.2. Dynamic LCE Model

This section mainly describes the dynamic model of light-driven contraction $\varepsilon_{L}\left(t\right)$ of the LCE balloon. We assume that the light-driven contraction strain $\varepsilon_{L}\left(t\right)$ is homogeneous in the thin LCE membrane of the balloon. For simplicity, the light-driven contraction $\varepsilon_{L}\left(t\right)$ is assumed to be proportional to the cis-isomers number fraction $\phi \left(t\right)$ of the LCE material, i.e.,

(7) $$\varepsilon_{L}\left(t\right)=-C_{0}\phi \left(t\right),$$

where $C_{0}$ is the contraction coefficient. In the following, we further provide the evolution law of the cis-isomers number fraction in Equation (7). 

The study by Yu et al. [66] found that the trans-to-cis isomerization of LCE could be induced by UV or laser with wavelength less than 400 nm. The number fraction $\phi \left(t\right)$ of the cis-isomer depends on the thermal excitation from trans to cis, the thermally driven relaxation from cis to trans, and the light driven relaxation from trans to cis. The number fraction $\phi \left(t\right)$ is governed by [53]

(8) $$\frac{\partial \phi }{\partial t}=\eta_{0}I_{0}\left(1-\phi \right)-\tau_{0}^{-1}\phi ,$$

where $\tau_{0}$ is the thermal relaxation time from cis to trans, $I_{0}$ is the light intensity, and $\eta_{0}$ is the light absorption constant. The number fraction $\phi \left(t\right)$ can be obtained by solving Equation (8) as

(9) $$\phi \left(t\right)=\frac{\eta_{0}T_{0}I_{0}}{\eta_{0}T_{0}I_{0}+1}+\left(\phi_{0}-\frac{\eta_{0}T_{0}I_{0}}{\eta_{0}T_{0}I_{0}+1}\right)\exp\left[-(\eta_{0}T_{0}I_{0}+1)\frac{t}{\tau_{0}}\right],$$

where $\phi_{0}$ is the number fraction of *cis-isomers* at the initial moment under illumination. 

In this paper, the LCE balloon switches between the illumination zone and the dark zone. For Case I that the LCE balloon is in the illumination zone with initial $\phi_{0}=0$, Equation (9) can be reduced to

(10) $$\phi \left(t\right)=\frac{\eta_{0}T_{0}I_{0}}{\eta_{0}T_{0}I_{0}+1}\left\{1-\exp\left[-(\eta_{0}T_{0}I_{0}+1)\frac{t_{1}}{\tau_{0}}\right]\right\}.$$

For Case II that the LCE balloon is in the illumination zone switched from the dark zone with transient $\phi_{0}=\phi_{\mathrm{dark}}$, Equation (9) can be reduced to

(11) $$\phi \left(t\right)=\frac{\eta_{0}T_{0}I_{0}}{\eta_{0}T_{0}I_{0}+1}+\left(\phi_{\mathrm{dark}}-\frac{\eta_{0}T_{0}I_{0}}{\eta_{0}T_{0}I_{0}+1}\right)\exp\left[-(\eta_{0}T_{0}I_{0}+1)\frac{t_{2}}{\tau_{0}}\right].$$

For Case III that the LCE balloon is in the dark zone ($I_{0}=0$) switched from the illumination zone with transient $\phi_{0}=\phi_{\mathrm{illum}}$, Equation (9) can be reduced to

(12) $$\phi \left(t\right)=\phi_{\mathrm{illum}}\exp\left(-\frac{t_{3}}{\tau_{0}}\right),$$

where $t_{1}$, $t_{2}$, and $t_{3}$ are the durations of current process, respectively. $\phi_{\mathrm{dark}}$ and $\phi_{\mathrm{illum}}$ are the number fractions of cis-isomers at the moment of switching from the dark zone into the illumination zone, and from the illumination zone into the dark zone, respectively. 

### 2.3. Solution Method

To conveniently investigate the dynamic jumping of LCE balloon, the dimensionless quantities are introduced as follows: ${\bar{I}}_{0}=\eta_{0}I_{0}\tau_{0}$, $\bar{t}=t/\tau_{0}$, $\bar{y}=y/r_{0}$, $\bar{r}=r/r_{0}$, $\bar{H}=H/r_{0}$, ${\bar{p}}_{\mathrm{am}}=2\pi p_{\mathrm{am}}\tau_{0}^{2}r_{0}/m$, ${\bar{n}}_{g}=3n_{g}RT\tau_{0}^{2}/2mr_{0}^{2}$, $\bar{E}=E_{\mathrm{eff}}V_{m}\tau_{0}^{2}/mr_{0}^{2}$, $\bar{\beta }=\beta \tau_{0}/m$, $\bar{g}=g\tau_{0}^{2}/r_{0}$, and ${\bar{F}}_{C}=F_{C}\tau_{0}^{2}/mr_{0}$. The governing Equations (1) to (3) can be rewritten in the dimensionless forms as

(13) $$-\bar{g}+{\bar{F}}_{C}-\bar{\beta }\dot{\bar{y}}=\ddot{\bar{y}},$$

(14) $${\bar{F}}_{C}=0\;\mathrm{for}\;\bar{y}\left(\bar{t}\right)>\bar{r}\left(\bar{t}\right),\;$$

(15) $${\bar{F}}_{C}=\frac{\left({\bar{n}}_{g}-{\bar{p}}_{\mathrm{am}}{\bar{r}}^{3}\right)\left({\bar{r}}^{2}-{\bar{y}}^{2}\right)}{2{\bar{r}}^{3}+\bar{r}\left({\bar{r}}^{2}-{\bar{y}}^{2}\right)\left(1-8\lambda \right)}\;\mathrm{for}\;\bar{y}\left(\bar{t}\right)\leq \bar{r}\left(\bar{t}\right),$$

where the constant λ can be rewritten as $\lambda =\frac{3{\bar{n}}_{g}}{16\left(\bar{E}+3{\bar{n}}_{g}\right)}$. The larger the value of ${\bar{n}}_{g}$ or the smaller the value of ${\bar{p}}_{\mathrm{am}}$, the greater the contact force ${\bar{F}}_{C}$ is, that is the easier the bounce is. 

Meanwhile, Equation (6) can also be rewritten as

(16) $${\bar{r}}^{3}+\frac{\bar{E}}{{\bar{p}}_{\mathrm{am}}\left(1+\varepsilon_{L}\right)}\bar{r}-\frac{\bar{E}+{\bar{n}}_{g}}{{\bar{p}}_{\mathrm{am}}}=0.$$

The discriminant of Equation (16) can be expressed as $\Delta =-\left(4p^{3}+27q^{2}\right)$, with $p=\frac{\bar{E}}{{\bar{p}}_{\mathrm{am}}\left(1+\varepsilon_{L}\right)}$ and $q=-\frac{\bar{E}+{\bar{n}}_{g}}{{\bar{p}}_{\mathrm{am}}}$. Since Δ<0, Equation (16) has only one real root, that is

(17) $$\bar{r}=\sqrt[{3}]{-\frac{q}{2}+\sqrt{\frac{q^{2}}{4}+\frac{p^{3}}{27}}}-\sqrt[{3}]{\frac{q}{2}+\sqrt{\frac{q^{2}}{4}+\frac{p^{3}}{27}}}.$$

From Equations (7) and (10)–(12), the light-driven contraction can be rewritten as follows, for Case I,

(18) $$\varepsilon_{L}\left(\bar{t}\right)=-\frac{C_{0}{\bar{I}}_{0}}{{\bar{I}}_{0}+1}\left\{1-\exp\left[-\left({\bar{I}}_{0}+1\right){\bar{t}}_{1}\right]\right\},$$

for Case II,

(19) $$\varepsilon_{L}\left(\bar{t}\right)=-\frac{C_{0}{\bar{I}}_{0}}{{\bar{I}}_{0}+1}-\left(\varepsilon_{\mathrm{dark}}-\frac{C_{0}{\bar{I}}_{0}}{{\bar{I}}_{0}+1}\right)\exp\left[-({\bar{I}}_{0}+1){\bar{t}}_{2}\right],$$

and for Case III,

(20) $$\varepsilon_{L}\left(\bar{t}\right)=-\varepsilon_{\mathrm{illum}}\exp\left(-{\bar{t}}_{3}\right),$$

where $\varepsilon_{\mathrm{dark}}$ and $\varepsilon_{\mathrm{illum}}$ are the light-driven contractions at the moment of switching from the dark zone into the illumination zone, and from the illumination zone into the dark zone, respectively. Since ${\bar{t}}_{1}$, ${\bar{t}}_{2}$, and ${\bar{t}}_{3}$ are the durations of current process, light-driven contraction $\varepsilon_{L}$ is process-related and time-dependent.

The initial conditions of the balloon can be given as

(21) $$\bar{y}={\bar{y}}_{0}\;\mathrm{and}\;\dot{\bar{y}}={\dot{\bar{y}}}_{0}\;\mathrm{at}\;\bar{t}=0.$$

Given the dimensionless parameters ${\bar{I}}_{0}$, $C_{0}$, $\bar{H}$, $\bar{\beta }$, $\bar{E}$, ${\bar{n}}_{g}$, ${\bar{p}}_{\mathrm{am}}$, $\bar{g}$, ${\bar{y}}_{0}$, and ${\dot{\bar{y}}}_{0}$, the solution of Equations (13)–(15) and (17)–(20)can be obtained numerically by programming in software Matlab based on the Runge–Kutta method. In the calculation, for the previous position ${\bar{y}}_{i-1}$ and light-driven contraction $\varepsilon_{L\left(i-1\right)}$, we can sequentially calculate the corresponding radius ${\bar{r}}_{i-1}$ from Equation (17) and contact force ${\bar{F}}_{C\left(i-1\right)}$ from Equation (14) or (15). We can further calculate the current position ${\bar{y}}_{i}$ from Equation (13) and the current light-driven contraction $\varepsilon_{Li}$ from Equations (18)–(20). Note that the LCE balloon is in the illumination zone while ${\bar{y}}_{i}>\bar{H}$, and in the dark zone while ${\bar{y}}_{i}<\bar{H}$. Next, based on this light-driven contraction $\varepsilon_{Li}$, we can further calculate the current radius ${\bar{r}}_{i}$ from Equation (17), and current contact force ${\bar{F}}_{Ci}$ from Equation (14) or (15) again. Then the current ${\bar{y}}_{i+1}$ and ${\bar{\varepsilon }}_{L\left(i+1\right)}$ can be sequentially calculated from Equations (13) and (18)–(20) again. By iteration calculation, we can obtain the time histories of light-driven contraction and position for the LCE balloon. 

## 3. Two Motion Regimes and Mechanism of the Self-Jumping

Based on the above governing equations, we numerically investigate the dynamics of the jumping balloon under steady illumination. We first present two typical motion regimes: the static regime and the self-jumping regime. Then, the corresponding mechanism of self-jumping is elucidated. 

### 3.1. Two Motion Regimes

To investigate the self-jumping of LCE balloon, we first need to determine the typical values of dimensionless parameters in the model. From available experiments [37,67,68], the material properties and geometric parameters are listed in Table 1. The corresponding dimensionless parameters are also listed in Table 2. In the following, these values of parameters are used to study the self-jumping of LCE balloon under steady illumination.

**Figure 2:**
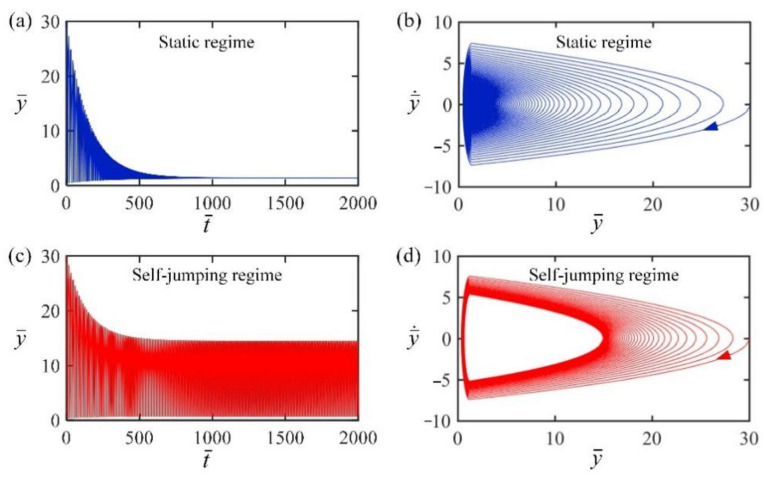
Time histories and phase trajectories for two motion regimes of the LCE balloon. (**a**,**b**) The static regime with ${\bar{I}}_{0}=0$; (**c**,**d**) the self-jumping regime with ${\bar{I}}_{0}=2$. The other parameters are $C_{0}=0.3$, $\bar{H}=4$, $\bar{\beta }=0.01$, $\bar{E}=500$, ${\bar{n}}_{g}=200$, ${\bar{p}}_{\mathrm{am}}=0.5$, $\bar{g}=1$, ${\bar{y}}_{0}=30$, and ${\dot{\bar{y}}}_{0}=0$. For the LCE balloon under steady illumination, there exist two typical motion regimes: the static regime and the self-jumping regime.

From Equations (13)–(15) and (17)–(20), the time histories and phase trajectories of light-powered jumping of the LCE balloon can be obtained. In the computation, we set $C_{0}=0.3$, $\bar{H}=4$, $\bar{\beta }=0.01$, $\bar{E}=500$, ${\bar{n}}_{g}=200$, ${\bar{p}}_{\mathrm{am}}=0.5$, $\bar{g}=1$, ${\bar{y}}_{0}=30$, and ${\dot{\bar{y}}}_{0}=0$. The numerical calculation shows that there exist two motion regimes of LCE balloon: the static regime and the self-jumping regime, as shown in Figure 2. For ${\bar{I}}_{0}=0$, the balloon with initial height first dropped because of gravity, and then hit and bounced from the rigid surface. Afterwards, the maximum height of the balloon gradually decreased due to air damping, and the balloon finally rested at the static equilibrium position, which is named as the static regime, as shown in Figure 2a,b. For ${\bar{I}}_{0}=2$, the maximum height of the balloon first decreased and then remained constant to a certain value as shown in Figure 2c,d. This result means that the balloon under steady illumination can jump continuously and finally develops into self-sustained jumping, which is named as the self-jumping regime. This is because that energy input transforming from light compensates the damping dissipation so as to maintain the self-jumping. In Section 3.2, the mechanism of self-jumping is explored in detail.

### 3.2. Mechanisms of the Self-Jumping

**Figure 3:**
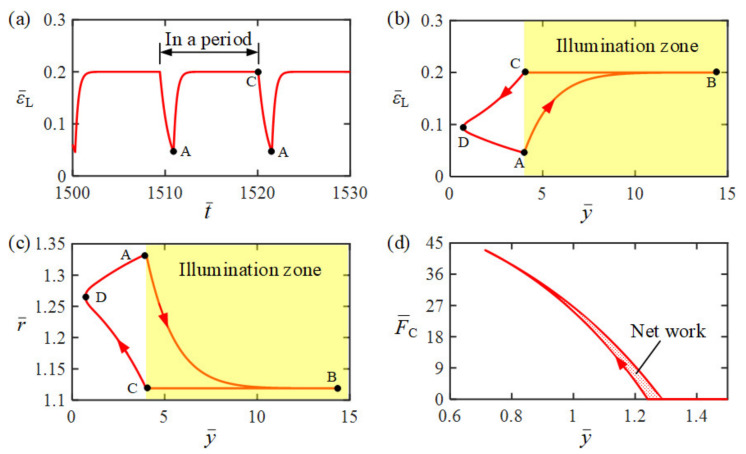
Mechanism of the self-jumping LCE balloon, for ${\bar{I}}_{0}=2$, $C_{0}=0.3$, $\bar{H}=4$, $\bar{\beta }=0.01$, $\bar{E}=500$, ${\bar{n}}_{g}=200$, ${\bar{p}}_{\mathrm{am}}=0.5$, $\bar{g}=1$, ${\bar{y}}_{0}=30$, and ${\dot{\bar{y}}}_{0}=0$. (**a**) The time history of light-driven contraction of the LCE balloon. (**b**) The dependence of light-driven contraction on the position of the balloon in one cycle of self-jumping. (**c**) The dependence of radius on the position of the balloon in one cycle of self-jumping. (**d**) The dependence of contact force on position. The dependence of both light-driven contraction and radius on position forms a cycle along the path of A→B→C→D→A. The area enclosed by the loop in Figure 3d represents the net work done by contact force, which compensates the damping dissipation and maintains the self-jumping.

To investigate the mechanism of self-jumping of LCE balloon, Figure 3 shows several key physical quantities of LCE balloon under steady illumination for the typical case in Figure 2c,d. Figure 3a shows the time history of light-driven contraction $\varepsilon_{L}$ of LCE balloon, presenting the characteristics of periodic changes over time. Figure 3b plots its dependence of light-driven contraction $\varepsilon_{L}$ on position $\bar{y}$ in one cycle of self-sustained jumping. The yellow shadow area in Figure 3 represents that the LCE balloon is in the illumination zone, and the curve A→B→C corresponds to the jumping process of the balloon in the illumination zone. When the LCE balloon jumps into the illumination zone, the light-driven contraction $\varepsilon_{L}$ gradually increases with time and tends to a limit value, as the curve A→B→C shown in Figure 3b. When the LCE balloon drops down into the dark zone, the light-driven contraction $\varepsilon_{L}$ undergoes a gradual decrease with time, as the curve C→D→A shown in Figure 3b. Therefore, the dependence of the light-driven contraction $\varepsilon_{L}$ on position $\bar{y}$ forms a cycle along the path of A→B→C→D→A.

**Figure 4:**
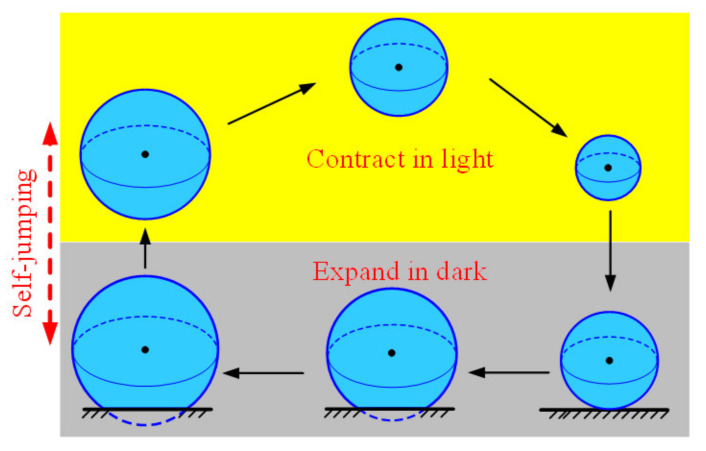
The process of the self-jumping LCE balloon in one cycle. The LCE balloon contracts in the illumination zone, while it expands in the dark zone. Due to the expansion of the balloon during the contact between the LCE balloon with rigid surface, the contact force does positive net work to maintain the self-jumping.

Similarly, Figure 3c plots the dependence of radius $\bar{r}$ on position $\bar{y}$ in one cycle of self-jumping. The radius $\bar{r}$ decreases in the illumination zone, while it increases in the dark zone. The dependence curve of radius $\bar{r}$ on position $\bar{y}$ forms a closed loop, as shown in Figure 3d. Further, Figure 3d plots the dependence of contact force ${\bar{F}}_{C}$ on position $\bar{y}$ during the contact of the LCE balloon with rigid surface. The contact force ${\bar{F}}_{C}$ of the balloon first increases and then decreases during the contact. Due to the expansion of the balloon during the contact in dark zone as shown in Figure 4, the dependence of contact force ${\bar{F}}_{C}$ on position $\bar{y}$ presents a closed clockwise curve in one cycle. The red shadow area in Figure 3d denotes the positive net work done by contact force ${\bar{F}}_{C}$, which compensates for the energy dissipation of damping; thus, the LCE balloon may continue jumping periodically under steady illumination.

## 4. Influence of System Parameters on the Self-Jumping

In this section, we investigate the trigger conditions for the self-jumping of LCE balloon, and the effects of various system parameters on frequency and amplitude. In this study, f denotes the dimensionless frequency, and A denotes the dimensionless amplitude which is the maximum value of position $\bar{y}$.

### 4.1. Effect of Initial Position

**Figure 5:**
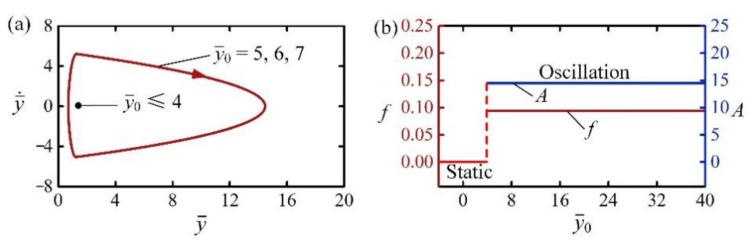
The effect of initial position on the self-jumping for $C_{0}=0.3$, ${\bar{I}}_{0}=2$, $\bar{H}=4$, $\bar{\beta }=0.01$, $\bar{g}=1$, ${\bar{n}}_{g}=200$, ${\bar{p}}_{\mathrm{am}}=0.5$, $\bar{E}=500$, and ${\dot{\bar{y}}}_{0}=0$. (**a**) Limit cycles. (**b**) Frequency and amplitude. There exists a critical ${\bar{y}}_{0}$ to trigger the self-jumping, and ${\bar{y}}_{0}$ does not affect the amplitude and frequency of self-jumping.

Figure 5 shows the effect of initial position ${\bar{y}}_{0}$ on the self-jumping of the LCE balloon. In the calculation, we set $C_{0}=0.3$, ${\bar{I}}_{0}=2$, $\bar{H}=4$, $\bar{\beta }=0.01$, $\bar{g}=1$, ${\bar{n}}_{g}=200$, ${\bar{p}}_{\mathrm{am}}=0.5$, $\bar{E}=500$, and ${\dot{\bar{y}}}_{0}=0$. Figure 5a plots the limit circles of the self-jumping balloon for different initial positions, in which there exists a critical position ${\bar{y}}_{0}$ about 4 for the phase transition between the static regime and the self-jumping regime. When the initial position is below the critical position, there is not enough energy input to compensate for the damping dissipation of the system, and thus the balloon develops into a static regime. For ${\bar{y}}_{0}=5$, ${\bar{y}}_{0}=6$, and ${\bar{y}}_{0}=7$, the self-jumping can be triggered and the limit circles are the same, as shown in Figure 5a. Figure 5b plots the frequency and amplitude of self-jumping as a function of the initial position ${\bar{y}}_{0}$, respectively. It can be easily observed that ${\bar{y}}_{0}$ does not change the amplitude and frequency of self-jumping. Considering that the parameter ${\bar{y}}_{0}$ can be transformed into the corresponding value ${\dot{\bar{y}}}_{0}$ through the energy transformation between gravitational potential energy and kinetic energy, it can be concluded that the initial conditions always have no effect on the amplitude and frequency of self-jumping, which is a general characteristic of self-oscillation [2].

### 4.2. Effect of Light Intensity

**Figure 6:**
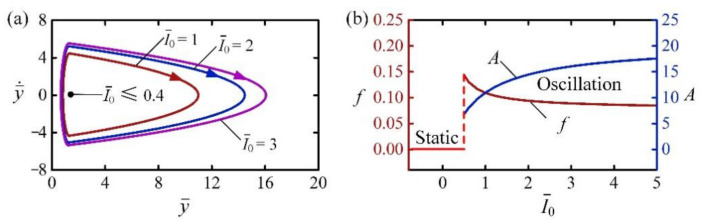
The effect of light intensity on the self-jumping, for $C_{0}=0.3$, $\bar{H}=4$, $\bar{\beta }=0.01$, $\bar{g}=1$, ${\bar{n}}_{g}=0.4$, ${\bar{p}}_{\mathrm{am}}=0.001$, $\bar{E}=500$, ${\bar{y}}_{0}=30$, and ${\dot{\bar{y}}}_{0}=0$. (**a**) Limit cycles. (**b**) Frequency and amplitude. With the increase of ${\bar{I}}_{0}$, the frequency of self-jumping decreases, while the amplitude increases.

Figure 6 describes the effect of light intensity ${\bar{I}}_{0}$ on the self-jumping of LCE balloon. In the calculation, we set $C_{0}=0.3$, $\bar{H}=4$, $\bar{\beta }=0.01$, $\bar{g}=1$, ${\bar{n}}_{g}=200$, ${\bar{p}}_{\mathrm{am}}=0.5$, $\bar{E}=500$, ${\bar{y}}_{0}=30$, and ${\dot{\bar{y}}}_{0}=0$. Figure 6a plots the limit circles of the self-jumping balloon for different light intensities, in which there exists a critical light intensity about 0.4 for triggering self-jumping. When the light intensity is less than the critical value, the energy input transforming from light cannot compensate for the damping dissipation, and the LCE balloon finally rests at the static equilibrium position. For ${\bar{I}}_{0}=1$, ${\bar{I}}_{0}=3$, and ${\bar{I}}_{0}=3$, the self-jumping is triggered and their limit circles are shown in Figure 6a. Figure 6b shows the effect of light intensity ${\bar{I}}_{0}$ on the frequency and amplitude of self-jumping. It can be seen that with the increase of ${\bar{I}}_{0}$, the frequency of self-jumping decreases while the amplitude increases. The exact reason for this is that the mechanical energy converted from light energy increases as ${\bar{I}}_{0}$ increases, and it takes a longer time for the balloon to jump to a higher position and drop down from this position.

### 4.3. Effect of Contraction Coefficient

**Figure 7:**
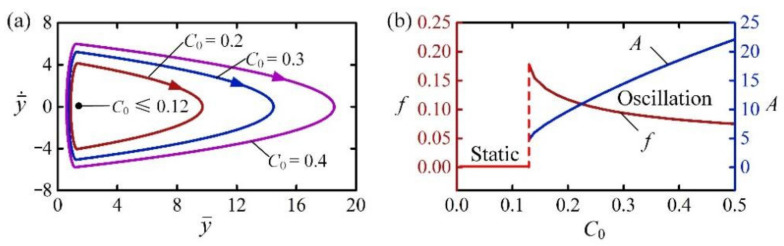
The effect of contraction coefficient on the self-jumping, for ${\bar{I}}_{0}=2$, $\bar{H}=4$, $\bar{\beta }=0.01$, $\bar{g}=1$, ${\bar{n}}_{g}=0.4$, ${\bar{p}}_{\mathrm{am}}=0.001$, $\bar{E}=500$, ${\bar{y}}_{0}=30$, and ${\dot{\bar{y}}}_{0}=0$. (**a**) Limit cycles. (**b**) Frequency and amplitude. With the increase of $C_{0}$, the frequency of self-jumping decreases while the amplitude increases.

Figure 7 shows the effect of contraction coefficient $C_{0}$ on the self-jumping of the LCE balloon. In the calculation, we set ${\bar{I}}_{0}=2$, $\bar{H}=4$, $\bar{\beta }=0.01$, $\bar{g}=1$, ${\bar{n}}_{g}=200$, ${\bar{p}}_{\mathrm{am}}=0.5$, $\bar{E}=500$, ${\bar{y}}_{0}=30$, and ${\dot{\bar{y}}}_{0}=0$. The critical contraction coefficient to trigger self-jumping is about 0.12. When the contraction coefficient is less than the critical value, the energy input from illumination is lower than the energy dissipation by damping, and the LCE balloon may stay at the static equilibrium position. For $C_{0}=0.2$, $C_{0}=0.3$, and $C_{0}=0.4$, the self-jumping is triggered and their limit circles are plotted in Figure 7a. Figure 7b shows the effect of contraction coefficient $C_{0}$ on the frequency and amplitude of self-jumping. With the increase of $C_{0}$, the frequency of self-jumping decreases while the amplitude increases. This result implies that increasing the energy input from light energy can increase both the jumping height and jumping time.

### 4.4. Effect of Dark Zone

**Figure 8:**
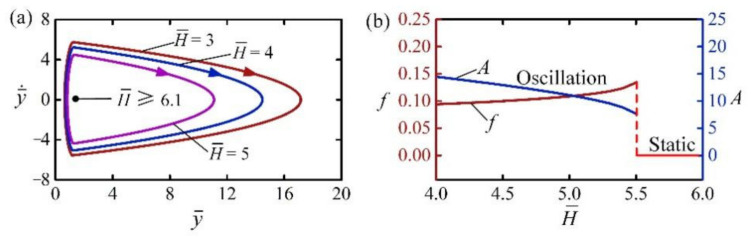
The effect of the dark zone on the self-jumping for ${\bar{I}}_{0}=2$, $C_{0}=0.3$, $\bar{\beta }=0.01$, $\bar{g}=1$, ${\bar{n}}_{g}=200$, ${\bar{p}}_{\mathrm{am}}=0.5$, $\bar{E}=500$, ${\bar{y}}_{0}=30$, and ${\dot{\bar{y}}}_{0}=0$. (**a**) Limit cycles. (**b**) Frequency and amplitude. With the increase of $\bar{H}$, the frequency of self-jumping increases while the amplitude decreases.

Figure 8 shows the effect of dark zone $\bar{H}$ on the self-jumping of the LCE balloon. In the calculation, we set ${\bar{I}}_{0}=2$, $C_{0}=0.3$, $\bar{\beta }=0.01$, $\bar{g}=1$, ${\bar{n}}_{g}=200$, ${\bar{p}}_{\mathrm{am}}=0.5$, $\bar{E}=500$, ${\bar{y}}_{0}=30$, and ${\dot{\bar{y}}}_{0}=0$. The critical dark zone for triggering self-jumping is about 6.1. For large $\bar{H}$, the LCE balloon eventually rests at the static equilibrium position. This is because that its light-driven contraction is almost fully recovered before impacting the rigid surface in the dark zone, and the energy dissipation by damping cannot be compensated by the positive net work of the contact force. For $\bar{H}=3$, $\bar{H}=4$, and $\bar{H}=5$, the self-jumping can be triggered and their limit circles are plotted in Figure 8a. Figure 8b shows the effect of the dark zone $\bar{H}$ on the frequency and amplitude of self-jumping. With the increase of $\bar{H}$, the frequency of self-jumping increases while the amplitude decreases. This result is because less energy is input into the system during the contact process for larger $\bar{H}$, the jumping amplitude of the balloon is smaller, and the corresponding frequency becomes larger.

### 4.5. Effect of Damping Coefficient

**Figure 9:**
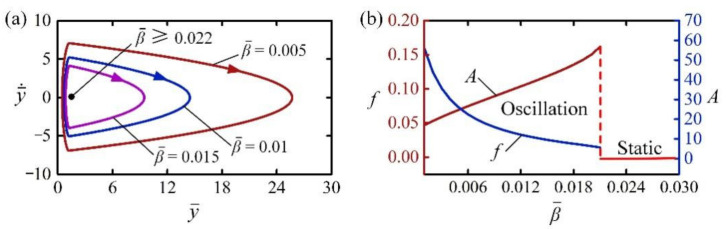
The effect of damping coefficient on the self-jumping for ${\bar{I}}_{0}=2$, $C_{0}=0.3$, $\bar{H}=4$, $\bar{g}=1$, ${\bar{n}}_{g}=200$, ${\bar{p}}_{\mathrm{am}}=0.5$, $\bar{E}=500$, ${\bar{y}}_{0}=30$, and ${\dot{\bar{y}}}_{0}=0$. (**a**) Limit cycles. (**b**) Frequency and amplitude. As the damping coefficient increases, the frequency increases while the amplitude decreases.

Figure 9 represents the effect of damping coefficient $\bar{\beta }$ on the self-jumping for ${\bar{I}}_{0}=2$, $C_{0}=0.3$, $\bar{H}=4$, $\bar{g}=1$, ${\bar{n}}_{g}=200$, ${\bar{p}}_{\mathrm{am}}=0.5$, $\bar{E}=500$, ${\bar{y}}_{0}=30$, and ${\dot{\bar{y}}}_{0}=0$. Figure 9a plots the limit cycles for different damping coefficients. Results indicated that there exists a critical $\bar{\beta }$ to trigger the self-jumping, which was numerically determined to be about 0.022. This is because that the energy input to the system cannot compensate the damping dissipation for $\bar{\beta }\geq 0.022$. For $\bar{\beta }=0.005$, $\bar{\beta }=0.01$, and $\bar{\beta }=0.015$, the self-jumping can be triggered and their limit circles are plotted in Figure 9a. The dependences of amplitude and frequency on damping coefficient are also shown in Figure 9b. As the damping coefficient increases, the frequency increases while the amplitude decreases. These results can also be explained by the energy competition between light energy input and damping dissipation. The larger the damping coefficient is, the more the energy dissipation generates, and thus the smaller the amplitude becomes.

### 4.6. Effect of Gravitational Acceleration

**Figure 10:**
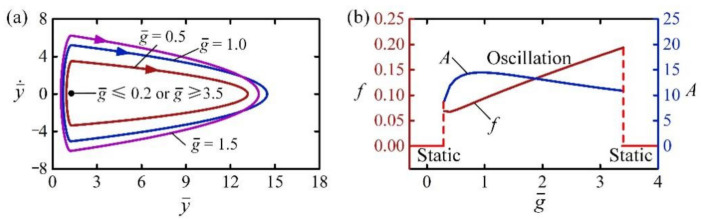
The effect of gravitational acceleration on the self-jumping for ${\bar{I}}_{0}=2$, $C_{0}=0.3$, $\bar{H}=4$, $\bar{\beta }=0.01$, ${\bar{n}}_{g}=200$, ${\bar{p}}_{\mathrm{am}}=0.5$, $\bar{E}=500$, ${\bar{y}}_{0}=30$, and ${\dot{\bar{y}}}_{0}=0$. (**a**) Limit cycles. (**b**) Frequency and amplitude. With the increase of $\bar{g}$, the frequency increases monotonically, while the amplitude first increases and then decreases.

Figure 10 reveals the effect of gravitational acceleration on the self-jumping for ${\bar{I}}_{0}=2$, $C_{0}=0.3$, $\bar{H}=4$, $\bar{\beta }=0.01$, ${\bar{n}}_{g}=200$, ${\bar{p}}_{\mathrm{am}}=0.5$, $\bar{E}=500$, ${\bar{y}}_{0}=30$, and ${\dot{\bar{y}}}_{0}=0$. Figure 10a plots the limit cycles of self-jumping of the LCE balloon for $\bar{g}=0.5$, $\bar{g}=1.0$ and $\bar{g}=1.5$. Careful calculation shows that for $0.2<\bar{g}<3.5$, the LCE balloon is in a self-jumping regime. The result can also be understood by the energy compensation between energy input and damping dissipation. For small $\bar{g}$, i.e., the light-driven contraction and deformation recovery are too fast, the LCE balloon rapidly recovers before contacting with the rigid surface, and does not expand during the contact. For large $\bar{g}$, i.e., the light driven contraction and deformation recovery are too slow, the LCE balloon barely expands during the contact with rigid surface. Therefore, the net work done by contact force is too small, and the energy input is unable to compensate for the energy dissipated by damping to maintain the self-jumping. Figure 10b shows the frequency and amplitude of self-jumping of the LCE balloon for $0.2<\bar{g}<3.5$, respectively. It can be observed that with the increase of $\bar{g}$, the frequency increased monotonically, while the amplitude first increased and then decreased.

### 4.7. Effect of Amount of Substance

**Figure 11:**
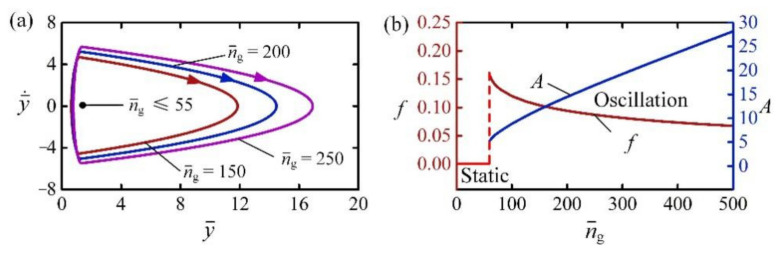
The effect of amount of substance on the self-jumping for ${\bar{I}}_{0}=2$, $C_{0}=0.3$, $\bar{H}=4$, $\bar{\beta }=0.01$, $\bar{g}=1$, ${\bar{p}}_{\mathrm{am}}=0.5$, $\bar{E}=500$, ${\bar{y}}_{0}=30$, and ${\dot{\bar{y}}}_{0}=0$. (**a**) Limit cycles. (**b**) Frequency and amplitude. With the increase of ${\bar{n}}_{g}$, the frequency of the self-jumping presents a downward trend, while the amplitude presents an upward trend.

Figure 11 shows the effect of amount of substance ${\bar{n}}_{g}$ on the self-jumping for ${\bar{I}}_{0}=2$, $C_{0}=0.3$, $\bar{H}=4$, $\bar{\beta }=0.01$, $\bar{g}=1$, ${\bar{p}}_{\mathrm{am}}=0.5$, $\bar{E}=500$, ${\bar{y}}_{0}=30$, and ${\dot{\bar{y}}}_{0}=0$. Figure 11a shows the limit cycles for different amounts of substance. There exists a critical ${\bar{n}}_{g}$ for the transition between static regime and self-jumping regime, which was numerically calculated to be about 55. For small ${\bar{n}}_{g}$, the contact force is also small as shown in Equation (15), and the net work done by the contact force is incapable of compensating for the damping dissipation to maintain the self-jumping. Figure 11b describes the dependences of frequency and amplitude on ${\bar{n}}_{g}$ for the self-jumping. It is clearly seen that with the increase of ${\bar{n}}_{g}$, the frequency of self-jumping presented a downward trend, while the amplitude presented an upward trend.

### 4.8. Effect of Ambient Pressure

**Figure 12:**
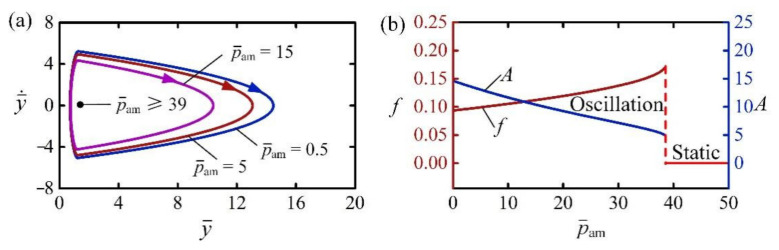
The effect of ambient pressure on the self-jumping, for ${\bar{I}}_{0}=2$, $C_{0}=0.3$, $\bar{H}=4$, $\bar{\beta }=0.01$, $\bar{g}=1$, ${\bar{n}}_{g}=200$, $\bar{E}=500$, ${\bar{y}}_{0}=30$, and ${\dot{\bar{y}}}_{0}=0$. (**a**) Limit cycles. (**b**) Frequency and amplitude. As ${\bar{p}}_{\mathrm{am}}$ increases, the frequency of self-jumping increases while the amplitude decreases.

Figure 12 presents the effect of ambient pressure ${\bar{p}}_{\mathrm{am}}$ on the self-jumping for ${\bar{I}}_{0}=2$, $C_{0}=0.3$, $\bar{H}=4$, $\bar{\beta }=0.01$, $\bar{g}=1$, ${\bar{n}}_{g}=200$, $\bar{E}=500$, ${\bar{y}}_{0}=30$, and ${\dot{\bar{y}}}_{0}=0$. From the limit cycles plotted in Figure 12a, there exists a critical ${\bar{p}}_{\mathrm{am}}$ for the trigger of self-jumping, which was numerically calculated to be about 39. This result means that the LCE balloon keeps in a static regime for ${\bar{p}}_{\mathrm{am}}\geq 39$, and a self-jumping regime for ${\bar{p}}_{\mathrm{am}}<39$. For large ambient pressure ${\bar{p}}_{\mathrm{am}}$, the net work done by contact force cannot compensate for the damping dissipation to maintain the self-jumping. This can be explained from Equation (15) that reducing ambient pressure has a similar effect to increasing the amount of substance ${\bar{n}}_{g}$. Figure 12b plots the frequency and amplitude of the self-jumping as a function of the ambient pressure ${\bar{p}}_{\mathrm{am}}$, respectively. It can be easily observed that with the increasing ${\bar{p}}_{\mathrm{am}}$, the frequency of self-jumping increased while the amplitude decreased.

### 4.9. Effect of Equivalent Elastic Modulus

**Figure 13:**
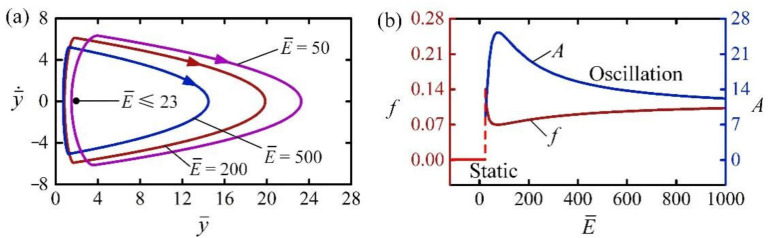
The effect of equivalent modulus on the self-jumping, for ${\bar{I}}_{0}=2$, $C_{0}=0.3$, $\bar{H}=4$, $\bar{\beta }=0.01$, $\bar{g}=1$, ${\bar{n}}_{g}=200$, ${\bar{p}}_{\mathrm{am}}=0.5$, ${\bar{y}}_{0}=30$, and ${\dot{\bar{y}}}_{0}=0$. (**a**) Limit cycles. (**b**) Frequency and amplitude. With the increase of $\bar{E}$, the frequency of self-jumping first decreases and then increases, while the amplitude first increases and then decreases.

Figure 13 shows the effect of equivalent modulus $\bar{E}$ on the self-jumping for ${\bar{I}}_{0}=2$, $C_{0}=0.3$, $\bar{H}=4$, $\bar{\beta }=0.01$, $\bar{g}=1$, ${\bar{n}}_{g}=200$, ${\bar{p}}_{\mathrm{am}}=0.5$, ${\bar{y}}_{0}=30$, and ${\dot{\bar{y}}}_{0}=0$. Figure 13a shows the limit cycles of self-jumping for different $\bar{E}$. The critical $\bar{E}$ for triggering the self-jumping regime was numerically calculated to be about 23. For small $\bar{E}$, i.e., the LCE balloon is very soft, both its contact force and net work done by the contact force are small during the contact; thus, the energy input is unable to compensate for the damping dissipation to maintain self-jumping. Figure 13b describes the dependences of frequency and amplitude on $\bar{E}$ for the self-jumping. It is obvious that with the increase of $\bar{E}$, the frequency of self-jumping first decreased and then increased, while the amplitude first increased and then decreased. This can be understood from the competition between the effects of $\bar{E}$ on radius increment and contact force during the contact. With the increase of $\bar{E}$, both the radius decrement in illumination zone and radius increment in dark zone decreased, while the contact force increased. Therefore, the net work done by the contact force during contact in one cycle first increases and then decreases.

## 5. Conclusions

Self-oscillation systems can maintain periodic motion upon constant stimulus, and have potential applications in the fields of autonomous robotics, energy-generation devices, sensors, mechano-logistic devices, and so on. Inspired by the active jumping of kangaroos and frogs in nature, we propose a self-jumping LCE balloon under steady illumination. Based on the balloon contact model and dynamic LCE model, a nonlinear dynamic model of self-jumping LCE balloon under steady illumination was formulated and numerically calculated by the Runge–Kutta method. The results indicated that there exist two typical motion regimes for the LCE balloon under steady illumination: the static regime and the self-jumping regime. Due to the expansion of the LCE balloon during the contact with a rigid surface in the dark zone, the positive net work can be done by the contact force in one cycle, and it can compensate for the damping dissipation to maintain self-jumping. The self-jumping of balloon can be triggered by controlling several key system parameters, including the light intensity, contraction coefficient, dark zone, amount of substance, equivalent elastic modulus, damping coefficient, and so on. In addition, the frequency and amplitude of self-jumping can also be controlled by these parameters. The self-jumping LCE hollow balloon with larger internal space has greater potential to carry goods or equipment, and may open a new insight into the development of mobile robotics, soft robotics, sensors, controlled drug delivery, and other miniature device applications.

## Figures and Tables

**Table 1:** Material properties and geometric parameters.

Parameter	Definition	Value	Units
$C_{0}$	Contraction coefficient	0.2~0.5	/
$\tau_{0}$	*trans*-to-*cis* thermal relaxation time	1~100	ms
$I_{0}$	Light intensity	0~1000	kW/m^2^
$\eta_{0}$	Light-absorption constant	0.0003	m^2^/(s∙W)
$r_{0}$	Reference radius of LCE balloon	0~5	mm
$V_{m}$	Volume of the LCE balloon	0~2	mm^3^
m	Mass of LCE balloon	0~2	mg
E	Elastic modulus of LCE balloon	1~10	MPa
$n_{g}$	Amount of substance of the gas	0~10^−7^	mol
R	Ideal gas constant	8.314	J/(mol·K)
$p_{\mathrm{am}}$	Ambient pressure	0~0.1	MPa
β	Damping coefficient	0~0.001	kg/s
g	Gravitational acceleration	10	m/s^2^

**Table 2:** Dimensionless parameters.

Parameter	${\bar{I}}_{0}$	${\bar{H}}_{0}$	${\bar{n}}_{g}$	${\bar{p}}_{\mathrm{am}}$	$\bar{\beta }$	$\bar{E}$	$\bar{g}$
Value	0~10^2^	0~20	0~10^4^	0~10^7^	0~50	10^2^~10^7^	10^−2^~10^2^

## Data Availability

The data that support the findings of this study are available upon reasonable request from the authors.
